# Graphene Hybrid Materials in Gas Sensing Applications †

**DOI:** 10.3390/s151229814

**Published:** 2015-12-04

**Authors:** Usman Latif, Franz L. Dickert

**Affiliations:** 1 COMSATS Institute of Information Technology, Department of Chemistry, Tobe Camp, University Road, 22060 Abbottabad, Pakistan; usmanlatif@ciit.net.pk; 2 Department of Analytical Chemistry, University of Vienna, Währinger Str. 38, A-1090 Vienna, Austria

**Keywords:** graphene, gas sensors, hybrid materials

## Abstract

Graphene, a two dimensional structure of carbon atoms, has been widely used as a material for gas sensing applications because of its large surface area, excellent conductivity, and ease of functionalization. This article reviews the most recent advances in graphene hybrid materials developed for gas sensing applications. In this review, synthetic approaches to fabricate graphene sensors, the nano structures of hybrid materials, and their sensing mechanism are presented. Future perspectives of this rapidly growing field are also discussed.

## 1. Introduction

Graphene is a monolayer of sp^2^-hybridized carbon atoms arranged in a honeycomb lattice which was discovered by Geim and Novoselov in 2004 [1,2]. Graphene shows excellent mechanical stability and unique electrical properties [3]. The perfect honeycomb lattice of graphene gives the highest mobility (~200,000 cm^2^/Vs) due to which the speed of electrons is around one million meters per second. Graphene is conductive, transparent and bendable, and is one of the strongest known materials [4]. Some of the important properties of graphene which make it useful for gas and vapor sensing are: its electron mobility at 300 K is 20,000 cm^2^/Vs, it has a resistivity of 10^−6^ Ω·cm, and its carrier density is 10^12^/cm^2^ [5].

Graphene is an attractive sensing material for fabricating a gas sensing device due to its intrinsic properties such as large surface area, higher interaction with gas molecules, and zero rest mass of its charged carriers [6]. The atoms in graphene sheets are considered as surface atoms providing large surface area per unit volume which makes them suitable for adsorption of gas molecules [7]. All atoms in graphene sheets are capable of interacting even with a single molecule of target gas [8]. This property makes graphene an ultrasensitive material for detecting gas molecules. The type of interactions between graphene atoms and adsorbing gas molecules vary from weak van der Waals interactions to strong covalent bonding which leads to a drastic change in electrical conductivity of graphene [9,10,11]. The change in electrical conductivity of graphene sheets upon exposure and subsequent adsorption of targeted chemical species lead to a change in free-electron concentration (increase/decrease if target species act as donor/acceptor) which can be monitored easily by a convenient electronic system. Graphene shows high carrier mobility at room temperature because of having zero rest mass of graphene carriers near its Dirac point [12]. Moreover, graphene has the lowest resistivity at room temperature among all the substances known so far.

This review article is intended to focus on the potential applications of graphene hybrids. It will bring forward most recent advancements in the field of graphene hybrid based gas sensors. Initially, we have included some papers in which graphene was utilized as sensing material to highlight its behavior towards different analytes, its high and low binding energy centers and some methods of its fabrication. Our main focus is on graphene hybrids which were divided into two types, *i.e*., hybrids of graphene with polymers and hybrids of graphene with metal/metal oxides.

## 2. Gas Sensors Based on Graphene

An epitaxial graphene film was grown on semi-insulating 4H silicon carbide (4H-SiC) wafer to fabricate nitrogen dioxide (NO_2_) gas sensor [13]. Epitaxial monolayer graphene is n-doped, and NO_2_ gas is a strong oxidizer which extracts electrons after its adsorption on a graphene surface. This effect reduces electron density in n-type material and leads to an increase in resistivity. Resistivity of the NO_2_ sensor device drastically changed after annealing at 120 °C which removes contaminants that remained on the chip after the fabrication process. There are two different types of adsorption centers on a graphene surface: one with high binding energy and the other is low binding energy centers. The annealing process increases sensor sensitivity towards NO_2_ down to sub-ppt level by activating high binding energy adsorption centers. Lower concentration of NO_2_ will occupy high binding energy adsorption centers which results in zero desorption due to strong binding of NO_2_ molecules. At higher concentration, desorption becomes significant which indicates adsorption centers with low binding energies are also occupied. Ozone treatment of graphene enhances its sensing behavior towards NO_2_ by introducing a number of functional oxygen groups on the surface [14]. Ko *et al*. developed a graphene sensor device by mechanical exfoliation of highly ordered pyrolytic graphite with subsequent deposition on silica/silicon (SiO_2_/Si) substrate [15]. The thickness of graphene deposited by the standard “scotch tape” method was in the range of 3.5–5 nm, which corresponds to 7–10 layers of graphene sheets. NO_2_ gas molecules adsorb/desorb on the graphene surface and there are three possible adsorption configurations: the nitro, nitrite and cyclo-addition configurations. The change or increase in graphene conductivity is attributed to the charge transfer between NO_2_ molecules and graphene where NO_2_ molecules act as an acceptor. The p-type semiconducting graphene was also grown by the ethanol-chemical vapor deposition (CVD) technique [16]. Nanomesh of monolayer graphene film was patterned by combining nanosphere lithography and reactive ion etching. This graphene nanomesh (GNM) was fabricated as gas sensor devices for detection of NO_2_ and ammonia (NH_3_) toxic gases at room temperature. The electron withdrawing effect of NO_2_ gas molecules will increase hole-density of p-type semiconductor graphene upon adsorption which leads to a decrease in resistance of sensor device. The responses of GNM were greater than their counterpart films which was attributed to the formation of a large number of edges in the structure that leads to hole doping. The resistance of GNM sensor increases when exposed to NH_3_ molecules. NH_3_ is an electron donor which results in depletion of holes in p-type GNM. Ethanol-based CVD grown graphene (G_ethanol_) expressed a p-type semiconducting behavior due to polycrystallinity in comparison to ambipolar electron transfer behavior of methanol-based CVD grown graphene (G_methanol_). The G_ethanol_ nanomesh contains highly disordered sp^3^ hybridization, a higher number of edge defects and functionalized oxygen groups affecting its electronic and transport characteristics in comparison to G_methanol_ nanomesh. The defects in G_methanol_ nanomesh were formed mainly due to edge formation on nanoholes whereas G_ethanol_ nanomesh contains additional unsaturated grain boundary of intrinsic topological defects due to its polycrystalline nature. These higher number of defects in G_ethanol_ nanomesh increase device sensitivity. The response times of both G_ethanol_ and G_methanol_ nanomesh devices are almost similar but response intensities are different. The defect sites in G_methanol_ nanomesh are less than G_ethanol_ nanomesh which leads to adsorption of a lower number of NO_2_ molecules.

A soft lithographical method was used to fabricate ammonia gas sensor by using highly oriented reduced graphene oxide (rGO) microbelts [17]. These uniformly structured microbelts have the advantage that they can be used in high numbers by utilizing a simple mask shielding method. An optical sensor for NH_3_ gas was fabricated on the basis of graphene/microfiber hybrid waveguide (GMHW) [18]. Mach-Zehnder interferometer (MZI) was utilized to spectrally demodulate the wavelength shift induced by NH_3_ adsorption on graphene. The graphene film was grown on copper (Cu) foil by CVD method with subsequent coating of polymethylmethacrylate (PMMA) film on graphene/Cu foil. The Cu foil was later on etched by ferric chloride (FeCl_3_) solution and PMMA/G was transferred onto magnesium fluoride (MgF_2_) substrate. Finally, the PMMA was removed by acetone. Then, a microfiber is attached onto graphene/MgF_2_ substrate by van der Waals and electrostatic forces. The nitrogen atom of NH_3_ becomes pentavalent after adsorption on graphene and captures two free electrons of two carbon atoms which leads to an increase in resistance of graphene. A hydrogen plasma was used to reduce graphene oxide (GO) at room temperature for fabricating the carbon dioxide (CO_2_) gas sensor [19]. The radicals and atoms in hydrogen plasma provide dissociation energies to oxygen functional groups for the reduction process. The reduction process enhanced sp^2^ crystalline interaction sites which in turn increases sensor sensitivity. The CO_2_ gas molecule acts as a donor and its adsorption on graphene sheet induces an increase in graphene conductance. Therefore, the physical adsorption of CO_2_ gas on graphene sheet is the dominant sensing mechanism which leads to a change in conductance of graphene sheet. The graphene samples were prepared by stamping method to fabricate CO_2_ gas sensor [20]. In this method, graphite flakes were deposited at specific locations on the substrate with a smaller amount of residue in comparison to the traditional method of mechanical cleavage in which “Scotch tape” was used. A thermally reduced GO was coated on a micro-hotplate by ac dielectrophoresis (DEP) to develop a chemo-resistive hydrogen (H_2_) gas sensor [21]. The GO was prepared by modified Hummer's method. All oxygen containing functional groups such as hydroxyl, epoxy, and carboxyl were removed during reduction of GO. An increase in resistance of rGO, a p-type semiconductor, was observed when exposed to H_2_ gas.

## 3. Gas Sensor Based on Graphene Polymer Hybrids

Seekaew *et al*. synthesized graphene-poly (3,4-ethylenedioxythiophene):poly (styrenesulfonate) (G-PEDOT:PSS) composite by gradually mixing graphene solution in PEDOT:PSS solution [22]. Screen printing technique was used to prepare interdigitated Ag electrodes on a flexible and transparent substrate. The G-PEDOT:PSS solution was inkjet printed on these electrodes by using Hewlett Packard (HP) deskjet 2000 printer with resolution of 1200 dots per inch (dpi) to fabricate the NH_3_ gas sensor. The sensitivity of the sensor device was assessed while being exposed to different concentrations of NH_3_ ranging from 5–1000 ppm. Whereas, selectivity of the sensor was investigated by using ethanol, methanol, toluene, acetone and diethylamine gases. The response of G-PEDOT:PSS sensor towards 500 ppm of NH_3_ was 9.6% which is higher than the responses of PEDOT:PSS (4.4%) and pristine graphene (2.4%) gas sensors. Similarly, the G-PEDOT:PSS hybrid sensor exhibited a response time of 3 min, which is fast in comparison to the PEDOT:PSS sensor.

Several sensing mechanisms have been proposed for sensors based on conducting polymers:

(i) Redox reactions
(ii) Charge transfer
(iii) Polymer swelling

(i) According to the first mechanism, the surface of G-PEDOT:PSS trap oxygen species (chemisorbed oxygen O^2−^) interact with NH_3_ molecules and release their electrons back to graphene/polymer composite. The holes of the valence band combine with electrons of the conduction band which results in lowering carrier concentrations and, finally, an increase in the resistance of the p-type G-PEDOT:PSS sensing film.

(ii) In the charge transfer process, the holes of conductive G-PEDOT:PSS surface interact with electron donating NH_3_ analyte after physisorption of NH_3_ molecules on the sensor surface. The delocalization degree of conjugated pi electrons of sensing films was increased, due to charge transfer from NH_3_ molecules, which leads to the formation of neutral polymer backbone. Thus, electrical conductivity of the sensing film decreases with a decrease in charge carriers.

(iii) A single PSS chain in PEDOT:PSS polymer film interacts with many PEDOT chains over its length. There is a very short interchain distance which favors the electron hopping process. However, this hopping process becomes difficult because of an increase in interchain distance of PEDOT due to swelling of the polymer with NH_3_ molecules’ diffusion. Graphene in G-PEDOT:PSS acted as a conductive pathway that favors the electron hopping process. The swelling process disrupts these conductive pathways as well as increases the PEDOT distance leading to a significant increase in resistance of the G-PEDOT:PSS sensor upon NH_3_ exposure. The resistance of graphene/polymer composite returns to its original value after NH_3_ desorption by dry air purging.

A surface plasmon resonance (SPR) based fiber optic gas probe was fabricated by utilizing a nanocomposite film based on PMMA and rGO [23]. The performance of the probe was tested against different gases like ammonia, chlorine, hydrogen, nitrogen, and hydrogen sulphide but the probe was more sensitive to ammonia gas. The GO was prepared by oxidation of graphite powder and it was chemically reduced by using hydrazine hydrate as a reducing agent to prepare rGO. The nanocomposite of PMMA-rGO was prepared by a bulk polymerization technique in which prepolymer MMA was polymerized in the presence of rGO.

The response mechanisms of fiber optic sensor based on PMMA-rGO nanocomposite for different gases can be explained as:

(i) The rGO surface contains polar oxygen functionalities which adsorb polar gas molecules and change its dielectric constant due to charge transfer; (ii) The incorporation of rGO into the polymer matrix creates conducting pathways, and adsorption of gas molecules may increase contact resistance between rGO sheets by increasing distance between rGO sheets. The change in volume of polymer matrix due to gas adsorption also increases the distance between sheets.

A self-assembly technique was used to create a conductive network of chemically reduced graphene oxide (CrGO) between parallel Au electrodes [24]. Au electrodes were modified with cysteamine hydrochloride to create a positive charge on electrode, which attracts negatively charged GO electrostatically. The GO sheets (assembled on Au electrodes) were directly reduced to rGO by hydrazine and pyrrole vapor to create a sensing platform for target gas molecules. The excellent sensing properties of rGO reduced by pyrrole in comparison to hydrazine reduced GO are attributed to its intrinsic properties as well as adsorbed polypyrrole (PPy) molecules. An ammonia gas sensor was prepared by reducing GO from pyrrole whereas GO was synthesized by a modified Hummers method [25]. During this reduction process, GO acted as oxidizing agent for oxidative polymerization of pyrrole. In this process, ethanol was used as a solvent which helps in evenly adsorbing PPy molecules on the rGO surface. Pyrrole reduced GO in the absence of ethanol was also produced for the sake of comparison. During this reduction process, the carboxyl (-COOH) groups had been removed from the surface of GO. The sensor devices were also prepared based on PPy nanofibers and rGO reduced by p-phenylenediamine (PPD). The sensor based on pyrrole reduced GO exhibited enhanced sensing behavior compared to PPy nanofibers alone or rGO produced via PPD. The better sensing performance of rGO device is due to the intrinsic properties of rGO sheets as well as adsorbed PPy molecules. The NH_3_ molecule is a reducing agent and has a lone pair of electrons which can be easily donated to p-type rGO that leads to an increase in sensor resistance. The response behavior of rGO sheets can be divided into two types: a fast response and a slow response. The fast response of rGO sheets is due to the gas adsorption onto binding sites with low energy (sp^2^-bonded carbon) and a slow response is due to high energy binding sites (e.g., vacancies, defects and oxygen functional groups). The sensing performance of this device also depends on the number of PPy molecules attached on the rGO surface. A lower number of PPy molecules on rGO surface allows complete interaction between NH_3_ molecules and sp^2^-bonded carbon atoms of rGO which leads to fast response of devices. A higher number of PPy molecules on rGO surface hinders the interaction between NH_3_ gas and sp^2^-bonded carbon, which severely affects the sensing performance.

Aniline was used to reduce GO in order to fabricate NH_3_ gas sensor [26]. rGO obtained in this process is attached to different states of polyaniline (PANI), *i.e*., acid-doped PANI attached rGO, dedoped PANI attached rGO and free rGO. The sensing properties of free rGO were much better than acid doped or dedoped rGO. Excellent sensing properties of completely free rGO based sensor is due to intrinsic properties of free rGO. The response of the sensor can be explained because of charge transfer from p-type rGO to adsorb ammonia molecules. In another study, a drop drying method has been utilized to produce ammonia gas sensor based on aniline reduced GO [27]. A conductive network of rGO sensing material is applied between electrode arrays. The rGO produced by aniline has enhanced sensing behavior in comparison to rGO reduced from hydrazine. The oxidized aniline (polyaniline (PANI)) attached to the rGO surface through π-π interaction and plays a key role in the sensing performance of the sensor device. The resistance of the sensor increases due to depletion of holes when electron donating NH_3_ gas interacts with the p-type graphene and causes charge transfer. PPy-rGO composite was prepared by a drop casting method to fabricate an ammonia gas sensor [28]. The composite based sensor was three times more sensitive than PPy thin film sensor and was capable of detecting a very low concentration (3 ppm) of ammonia. However, the recovery of PPy-rGO composite sensor is very difficult due to the presence of high energy binding sites.

A chemiresistor sensor for NO_2_ gas molecules was fabricated by a porous conducting polymer PEDOT nanocomposite prepared on rGO film [29]. The increase in charge carriers of composite was attributed to π-π interactions between PEDOT and rGO sheets which leads to a decrease in composite resistance. Adsorption of NO_2_ gas molecules increases the number of charge carriers and tremendously decreases resistance of rGO-PEDOT composite.

**Figure 1:**
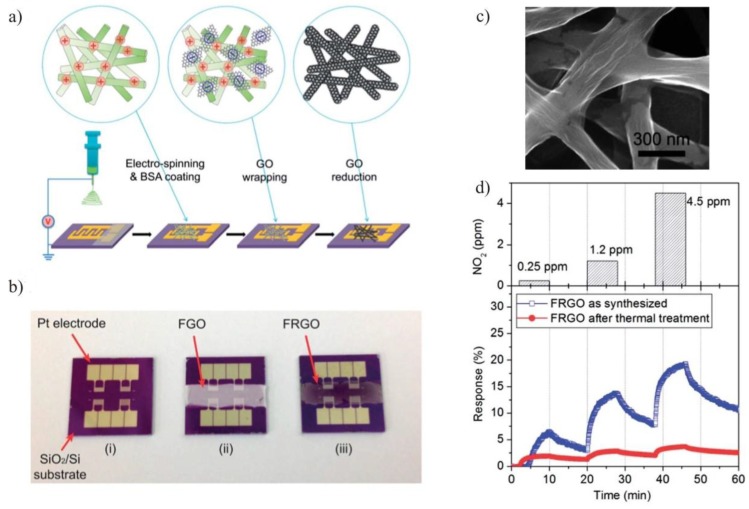
(**a**) Schematic representation of fabricating a gas sensor based on rGO/nylon-6 nanofibers; (**b**) Optical images of the bare device (i), GO/nylon-6 coated, formation of GO network based on interwoven fibers (FGO) (ii), and rGO/nylon-6 sensing device, rGO gas sensors fabricated on electrospun nanofibers (FRGO) (iii); (**c**) SEM image of rGO nanofiber; (**d**) Sensing response of the devices based on FRGO as synthesized (blue line) and thermally treated FRGO at 300 °C (red line). {Reprinted with permission from [30], ©2014 Royal Society of Chemistry}.

A combination of an electrospinning method and GO wrapping on nylon-6 fibers through an electrostatic self-assembly, followed by a low-temperature chemical reduction, was utilized to produce a sensor for detecting NO_2_ gas. A procedure to fabricate rGO/nylon-6 nanofiber mats (FRGO) based gas sensor has been illustrated in Figure 1a and Figure 1b. Nylon-6 nanofiber scaffold was produced by using electrospinning method directly onto SiO_2_/Si substrate having platinum (Pt) interdigitated electrode (IDE) arrays [30]. These nanofibers were functionalized using bovine serum albumin (BSA) molecules which induce a positive charges on nanofiber surface and improves GO adsorption onto electrospun nanofibers. The GO sheets are negatively charged and form a uniform coating on positively charged BSA functionalized nylon-6 nanofibers via electrostatic self-assembly. Finally, a low temperature chemical reduction method was utilized to reduce GO/nylon-6 nanofiber mats. SEM image of FRGO shows that porous interwoven structure of nylon-6 nanofibers wrapped completely with rGO sheets as shown in Figure 1c. The FRGO based NO_2_ gas sensor exhibited a sensor response of 7% to 0.25 ppm of NO_2_ as shown in Figure 1d. The excellent sensing performance of GO/nylon-6 nanofiber mat was attributed to the large surface area of nanofibers. This hybrid nanofiber has approximately π (~3.14) times’ higher surface area than flattened fiber, which leads to better adsorption and, finally, higher sensing performance of the sensor.

The GO was reduced to rGO during the formation of PPy nanofibers by utilizing a one-step redox reaction under UV illumination at room temperature [31]. The rGO-PPy nanofiber composite is highly sensitive to NO_2_ gas. The pyrrole monomer was absorbed by the GO surface by π-π stacking interactions. Polymerization of pyrrole to PPy and reduction of GO to rGO occur simultaneously under UV illumination. Graphene oxide (GO) was synthesized by acid dissolution method in which graphite powder is heated with sulfuric acid and phosphoric acid in the presence of potassium permanganate. Then, prepared GO was chemically reduced with ascorbic acid to synthesize rGO.

In another study, a paper-like nanocomposite of G/nylon-6 was fabricated by incorporating rGO onto a nylon-6 (N6) membrane via vacuum assisted self-assembly (VASA) method [32]. The dispersion of rGO onto a nylon sheet creates a conducting network and resistance to polymer nanocomposite decreases with an increase in rGO dispersion. There is an abrupt increase in electrical resistance of graphene nanocomposite when exposed to trimethylamine (TMA) concentrations. The GO was prepared by modifying Hummers method in which graphite was oxidized with concentrated sulfuric acid [33]. Then, CrGO was prepared by reducing GO with PPD. The synthesis of CrGO via GO reduction eliminates many of the oxygen-containing functional groups (restoring original properties of sheets) but it still contains oxygen-based moieties and structural defects. The p-type semiconducting behavior of resultant CrGO is attributed to the existing oxygen-based moieties and structural defects which are electron withdrawing and promote holes in the valence bond of CrGO which play a key role in sensing properties. The analyte dimethyl methylphosphonate (DMMP) is a strong electron donor which depletes holes from the valence bond of CrGO, resulting in an increase in resistance after being adsorbed on the CrGO surface.

## 4. Gas Sensors Based on Metal/Metal Oxide Graphene Hybrid

Hong *et al*. deposited palladium nanoparticles (Pd NPs) on single-layer graphene (SLG) via galvanic displacement between graphene-buffered Cu and Pd ion followed by spin-coating with a PMMA membrane layer to fabricate a hydrogen gas sensor [34]. This process involved four main steps: growth of SLG on Cu foil by CVD method, deposition of Pd NPs on SLG via a galvanic displacement reaction, transfer of Pd NP/SLG hybrid onto electrode-patterned glass substrate, spin coating of PMMA polymer to fabricate PMMA/Pd NPs/SLG hybrid sensor. The mechanism of H_2_ detection can be explained by dissolution and dissociation of H_2_ molecules into atomic hydrogen in the presence of Pd NPs deposited on graphene. Pd-hydrides (PdHx) were formed (as a function of Pd is diminished) when H_2_ molecules dissolved into atomic hydrogen after being adsorbed on Pd NPs surfaces. The resistance of the sensor increases as the diminished function of PdHx results in increased electron transfer to graphene from PdHx. Increase in the density of Pd NPs on SLG increases PdHx formation due to the high surface area of Pd. This will change the conduction pathways from graphene to Pd film like structures, which decreases gas response sensitivity. Pak *et al*. synthesized palladium-decorated graphene nanoribbons (Pd-GNRs) for detecting hydrogen gas sensor [35]. Laser interference lithography was utilized to prepare polymer residue-free GNRs. These nanoribbons were further decorated with Pd NPs. The GNRs were used for enhancing electrically conductive pathways, and Pd NPs were utilized as catalysts for sensing hydrogen gas. The SLG was grown on Cu/Ni film coated on a SiO_2_/silicon wafer by inductively coupled plasma chemical vapor deposition (ICP-CVD) technique. The CVD-grown graphene was transferred to desired substrate by following polymer PMMA transferring technique. A periodically aligned GNR array was synthesized by placing a chromium interlayer under photoresist via laser interference lithography and then, incorporating it into the Pd-decorated hydrogen sensors. The developed sensor exhibited excellent response towards H_2_ gas with response/recovery time of 60 s/90 s.

Another hydrogen gas sensor has been fabricated by incorporating Pd-WO_3_ (Palladium-tungsten trioxide) nanostructures in GO and partially reduced graphene oxide (PrGO) by utilizing a controlled hydrothermal process [36]. The homogeneous and stable WO_3_ sol was synthesized from sodium tungstate powder Na_2_WO_4_ and its ribbon like nanostructure was formed by adding K_2_SO_4_. The GO was synthesized by following Hummer’s method, whereas PrGO suspension was obtained through the hydrothermal method. Pd-WO_3_/GO and Pd-WO_3_/PrGO hybrid was produced by adding Pd-WO_3_ drop by drop to GO and PrGO suspensions under vigorous stirring. In this hybrid structure, Pd NPs and adsorbed oxygen species (such as O^−^, O_2_^−^, O^2−^) play a dominant role in sensing target gas molecules. These oxygen species capture electrons from conduction band of WO_3_ (as an n-type semiconductor) which generate a depletion layer with low electrical conductivity. Hydrogen as a reducing gas remove oxygen charge after interacting with these oxygen species and the generation of electrons in this reaction results in conductivity increase. The rate of this reaction increases in Pd NPs’ presence due to molecular hydrogen dissociation into active atoms. PdHx hybrids are also formed due to hydrogen dissolution in Pd, which has diminished function. This effect also leads to donation of electrons to the conduction band of WO_3_ and increases the electrical conductivity of Pd-WO_3_/graphene hybrid.

Phan *et al*. synthesized graphene-supported platinum/palladium (Pt/Pd) core-shells hybrid to fabricate a hydrogen sensor device [37]. At first, Pt/Pd core-shells were prepared by using highly uniformed colloidal solution of Pd nanocubes as the core and, then, Pt was coated on Pd cubes as shells. In the second step, graphene was decorated with these Pt/Pd core-shells to synthesize a Pt/Pd core–shell graphene hybrid. The sensor response of graphene hybrid towards 10,000 ppm (1% H_2_) is 36% at room temperature with an excellent response/recovery time of 3/1.2 min whereas the detectable range of Pt/Pd core-shell hybrid is 1–40,000 ppm. Another hydrogen sensor was fabricated by using Pt and platinum-iron alloy (Pt_3_Fe) nanoparticles which were decorated on nitrogen doped graphene [38]. GO was prepared from graphite powder and, then, converted to graphene by hydrogen exfoliation at high temperatures. PSS served as anionic electrolyte and pyrrole monomer as a nitrogen source for incorporating nitrogen functional groups in graphene. The defects introduced to graphene by incorporating nitrogen in the structure facilitate uniform dispersion of Pt and Pt_3_Fe alloy nanoparticles. Then, ethylene glycol reduction technique was followed to decorate graphene with Pt and Pt_3_Fe alloy nanoparticles. Chloroplatinic acid (H_2_PtCl_6_) solution was used for decorating graphene with Pt nanoparticles whereas a mixture of H_2_PtCl_6_ and FeCl_3_ solutions was used for Pt_3_Fe alloy decoration. The sensor sensitivities were measured through exposure to 1%–4% of H_2_ which indicates that sensitivity decreases when alloying Pd with 3D transition metals.

Anand *et al*. synthesized a nanocomposite of graphene and zinc oxide (ZnO) by *in situ* reduction of zinc acetate and GO during refluxing whereas GO was prepared from graphite powder by following Hummer’s method [39]. A paste of G-ZnO was prepared by mixing it with ethanol, then a thick film of this paste was coated on alumina substrate having gold electrodes to fabricate the H_2_ sensor. A number of G-ZnO thick film sensors were fabricated by varying the graphene content (0.6, 0.9, 1.2 and 1.5 wt%) in G-ZnO composite. The G-ZnO composite having 1.2 wt% of graphene content is very sensitive in comparison to ZnO (optimum operating temperature 400 °C) and other composites, and gives a sensor response of 3.5 at an optimum operating temperature of 150 °C. The composites exhibited improved performance even at lower temperatures in comparison to bare ZnO. Mishra *et al.* synthesized rGO decorated with tin oxide (SnO_2_) quantum dots (QDs) by a surfactant assisted hydrothermal method [40]. Graphite powder was used for synthesizing rGO and SnO_2_ QDs were prepared from SnCl_4_·5H_2_O by a hydrothermal method. The rGO-SnO_2_ QDs based sensor was used to detect H_2_ and liquefied petroleum gas (LPG) in a concentration range of 100–500 ppm and it gives a response of 89.3% to H_2_ (500 ppm) and 92.4% to LPG (500 ppm) at an operating temperature of 200 °C and 250 °C, respectively.

Multiple gas sensors based on Pt decorated rGO nanostructures were fabricated via DEP. Different parameters of the DEP technique such as applied voltage, frequency and processing time were optimized to assemble Pt-rGO nanocomposite into the electrodes [41]. For this purpose, Pt nanoparticles solution was mixed with GO nanostructure solution and then dropped into the gaps of electrodes. An AC voltage was applied which pulled and bound nanohybrid structures to the substrate surface between electrodes under the influence of electric field gradient. A sensor device based on a DEP assembled rGO structure without Pt was also fabricated for comparison. The sensitivities of both these sensor devices were investigated through exposure to different concentrations (200–1000 ppm) of H_2_, NH_3_, and NO gases at room temperature. The Pt nanoparticles decorated rGO sensor device exhibited an improvement of 100%, 60% and 25% to H_2_, NH_3_, and NO gases in comparison to the sensor having an rGO structure without Pt.

A surface plasmon resonance based gas sensor was synthesized depositing graphene flakes over gold (Au) nanoparticles [42]. These nanoparticles were chemically attached to a functionalized fused silica substrate. The sensing performance depends on the concentration of Au NPs in rGO coupled Au thin films. A higher concentration of Au NPs in rGO-Au thin films leads to the best sensing performance towards H_2_, NO_2_, and CO. The rGO coupled Au NPs’ thin film sensor was exposed to 1 ppm of NO_2_, 10,000 ppm and 100 ppm of H_2_ and 10,000 ppm of CO. A change in optical properties was observed while exposing the sensitive surface (graphene flakes-Au NPs) to different gases such as reducing and oxidizing gases. This shift in surface plasmon resonance is attributed to an electron transfer to Au NPs and sp^2^-hybridized carbons atoms of graphene oxide.

A chemiresistive type of acetylene gas sensor was synthesized based on silver (Ag) loaded ZnO-rGO hybrid via a chemical route [43]. A chemiresistive semiconductor sensor was based on the change in resistance of sensing material after interacting with adsorbed gas. The sensing mechanism can be described in terms of oxygen adsorption reaction on the sensing material surface, which depends on operating temperature, and stable oxygen species are O_2_^−^, O^−^, O^2−^ that operate below 100 °C, between 100 and 300 °C and above 300 °C, respectively. The oxygen adsorption reactions can be represented as follows:

$$O_{2\;}\left(gas\right)\;+\;e^{-}\;\to \;O_{2}^{-}\;\left(low\;temperature\right)$$

$$O_{2}\left(gas\right)\;+\;2e^{-}\;\to \;2O_{ads}^{-}\;\left(moderate\;temperature\right)$$

$$O_{2}\left(gas\right)\;+\;4e^{-}\;\to \;2O_{ads}^{2-}\;\left(high\;temperature\right)$$

Non-ZnO modified graphene exhibited p-type behavior, whereas, in case of ZnO modified graphene, an opposite sensitivity response was observed due to n-type sensitivity nature of ZnO. Ag has better oxygen dissociation ability than ZnO and activates molecular oxygen dissociation on hybrid surface and creates more acetylene sensing active sites on the surface. The oxygen species on sensing material becomes more active with acetylene molecules, which donate electrons to the sensing surface.

A room temperature acetone gas sensor based on SnO_2_-rGO hybrid composite film was fabricated on a printed circuit board (PCB) substrate (having interdigitated electrodes) by utilizing a hydrothermal method [44]. It was prepared by reacting SnCl_4_ with rGO and, later on, autoclave at high temperature. Then, the sensor was fabricated on PCB by microfabrication and sensing film was casted by drop casting. The higher sensing performance of SnO_2_-rGO hybrid composite can be attributed to three factors:

(i) Incorporation of SnO_2_ nanoparticles in rGO creates a porous nanostructure by reducing aggregation of rGO sheets. The porous structure has greater surface area and more active sites such as defects, vacancies, oxygen functional groups and sp^2^-bonded carbons which adsorb and diffuse acetone gas molecules.

(ii) The gas sensing performance is due to heterojunction formation at the interface between n-type SnO_2_ nanoparticles and p-type rGO nanosheets. The electron donor ability of acetone gas increases negative charge carriers in n-type thin film and reduces an overall sensor resistance.

(iii) The surface oxygen species such as O^2−^ is also responsible for gas sensing. These chemisorbed oxygen molecules are ionized to oxygen species by capturing free electrons from SnO_2_ nanoparticles. The rGO exhibits p-type semiconducting behavior and contains dominantly positive charge carriers (holes). The adsorbed acetone gas is an electron donor which changes the concentration of holes in rGO, resulting in a change in rGO resistance. The SnO_2_-rGO hybrid composite shows a higher response to acetone gas, which is attributed to high surface area and porous nanostructure. 

A thin film of zinc oxide was deposited on graphene film by Atomic Layer Deposition (ALD), whereas graphene film was grown by CVD [45]. A ZnO/G hybrid sensor was evaluated by exposing formaldehyde vapors. ALD is better than CVD because its synthesized layer is uniform, conformal and atomically precise and it also does not damage the substrate due to its low temperature and chemisorptions processing. Non-ZnO modified graphene exhibited p-type behavior and shows an increase in resistance upon exposure to reducing gas formaldehyde, which donates its electrons to graphene. Whereas, in case of ZnO modified graphene, an opposite sensitivity response was observed due to the n-type sensitivity nature of ZnO. The oxygen molecules adsorbed on ZnO extract electron from the conduction band of ZnO and form O^−^, O_2_^−^, O^2−^ which leads to a formation of depletion layer and, finally, increases ZnO resistance. The adsorbed oxygen species interacts with formaldehyde and releases trapped electrons back to ZnO conduction band resulting in a decrease in sensor resistance. Thus, ZnO-G hybrid exhibits enhanced sensor response in comparison to ZnO or graphene alone as a sensing material.

A graphene-zinc ferrite (G-ZnFe_2_O_4_) composite was prepared by solvothermal method and utilized to fabricate acetone gas sensor [46]. The graphene for this composite was prepared by reduction of graphene oxide with hydrazine hydrate whereas graphene oxide was synthesized by modified Hummer’s method. ZnFe_2_O_4_ was produced by using Zn(NO_3_)_2_·6H_2_O and Fe(NO_3_)_3_·9H_2_O reagents as precursors. Acetone molecules interact with oxygen species on sensor surface and oxidize them to carbon dioxide and water along with release of free electrons, which results in a decrease in sensor resistance. A gas sensor was fabricated composed of ZnO conductive bottom layer on a metal foil (vertically aligned ZnO nanorod channel) and a graphene based top conductive electrode [47]. A conductive layer of ZnO nanorods was prepared on stainless steel foil by following a wet hydrothermal method. The graphene film was grown by CVD method. The change in conductance is based on the depletion of ZnO NR channels by oxygen ionosorption on their surface by either O^−^ or O_2_^−^ depending on operating temperature. The interaction of reducing gas ethanol with ZnO NR substitutes surface-bound oxygen which releases electrons back to ZnO crystals and leads to an increase in electrical conductivity. The higher sensitivity of this sensor is attributed to the existence of pores between monolithic nanorods which means more surface area and greater sensor response.

Khadem *et al*. synthesized G-ZnO hybrid to fabricate a gas sensor [48]. ZnO nanowires in this hybrid has two important roles: reduction of GO to obtain graphene as well as acting as an efficient electromechanical actuator due to their piezoelectric properties. GO was prepared by utilizing Hummer’s method in which graphite powder was oxidized to produce GO. Laterally grown ZnO nanowires (NWs) were formed on Si wafer as substrate. The synthesized GO sheets were dispersed on previously grown ZnO NWs by dip-coating. The ZnO NWs have been used to reduce GO sheets through exposure to UV illumination. The gas sensing behavior of ZnO-G was evaluated through expoosure to ethanol vapors. The conductivity of ZnO NWs increases upon ethanol (which behaves as donor) exposure because of ethanol interaction with oxygen vacancies in ZnO NWs. The enhanced sensitivity of the ZnO-G hybrid structure in comparison to ZnO was attributed to the greater surface area of graphene. Moreover, sp^2^ orbitals of carbon in graphene act as active sites which interact with gas molecules and change their conductivity according to the electron affinity of gas molecules as well as number of adsorbed gas molecules.

In a comparison of G-mica and G-SiO_2_ based ammonia gas sensors, graphene is supported on two different substrates. Mica substrate induces more p-doping in graphene which leads to enhanced sensitivity [49]. Muscovite mica is a well-known, natural insulating material having the formula KAl_2_(Si_3_AlO_10_)(FOH)_2_ and possessing high dielectric strength and chemical and thermal stability. Monolayer graphene (MLG) was prepared by CVD on Cu foil and, subsequently, transferred to the desired substrate by a PMMA assisted transfer technique. NH_3_ molecules have strong electron donating capability and donate electrons to p-type graphene when adsorbed. The effect will shift Fermi level to Dirac point and leads to an increase in resistance upon exposure to NH_3_ gas. The higher sensitivity on mica substrate can be explained in terms of substrate dependant doping and hydrophilicity. The mica substrate shift graphene Fermi level to downward which leads to form additional states around Fermi level. Thus, graphene orbitals significantly overlap with highest occupied molecular orbitals (HOMO) of NH_3_ and cause large charge transfer. A gas sensor has been fabricated by using SnO_2_-G composite which was synthesized by one-pot method with GO and SnCl_2_ as precursors [50]. The SnO_2_ was n-type semiconductor and its interaction with reducing/oxidizing gas molecules leads to an increase/decrease in conductivity, respectively. The SnO_2_-G composite also exhibited increased conductivity while in contact with reducing gases such as hydrogen sulfide (H_2_S) and NH_3_.

Xiang *et al*. synthesized a composite of PPy and graphene nanoplatelets (GNs) by chemical polymerization and decorated it with titanium dioxide nanoparticles (TiO_2_ NPs) by sol-gel technique (TiO_2_@PPy-GN) [51]. The TiO_2_ NPs were in the range of 10–30 nm which were well dispersed on the PPy-GN composite. NH_3_ sensor was fabricated by drop-coating a suspension of TiO_2_@PPy-GN in DMF on integrated indium-tin oxide (ITO) electrodes. The sensors of GN, PPy-GN thin films were also fabricated for comparison. TiO_2_@PPy-GN sensor content showed higher sensitivity to NH_3_ and faster response and recovery times in comparison to GN and PPy-GN thin films. The response of TiO_2_@PPy-GN sensor is about 12 and 4.5 times higher in comparison to GN and PPy-GN composite sensors. In another study, a single step synthetic procedure was applied to fabricate a room temperature gas sensor for CO, NH_3_ and NO gases based on photoluminescence (PL) properties of zinc oxide (ZnO) decorated graphene oxide sheets (GO) as sensitive coatings [52]. GO was synthesized by following Hummer's method and lithium hydroxide was utilized for reducing GO to produce rGO. ZnO nanoparticles were produced by using zinc acetate as precursor. The ZnO based gas sensors have high sensitivity and moderate selectivity which can be further improved by doping but these sensors work at high temperature. Whereas, the sensors based on graphene or CNTs can work at room temperature but have poor selectivity.

A hybrid of rGO and cobalt hydroxide (Co(OH)_2_) nanoflakes were prepared by one-pot reflux method to fabricate NOx gas sensors [53]. The negative charges on graphene sheets (such as hydroxyl, carbonyl, and epoxy groups) interact strongly with positive ions such as Co^2+^ in an aqueous solution through electrostatic interaction to form Co(OH)_2_ nanoparticles on graphene sheets. The small nanocrystals with high energy recrystallize to form nanosheets and nanopetals, in a manner of energy minimization. The nanopetals arrange themselves to form nanoflowers. Some of the nano-nuclei form the shape of nanowires because of time and space constraints. Gas-sensing performance of this hybrid can be explained as: the chemisorbed oxygen present on Co(OH)_2_-rGO hybrid interact with NO_x_ molecules which extract electrons from hybrid, leading to an increase of hole conductance, which finally decreases the resistance of p-type hybrid. The oxygen adsorption plays an important role in the gas sensing mechanism of the Co(OH)_2_-rGO composite sensor which follows the surface charge model. The adsorption of NO_x_ and high electron affinity of NO_x_ molecules lead to electron transfer from the Co(OH)_2_-rGO layer to NO_x_. Thus, adsorption of NO_2_ on graphene hybrid converts NO_2_ to NO_2_^−^ and NO to NO^−^. The target gas molecules NO or NO_2_ also adsorbed onto graphene hybrid and reacted with O^-^ to generate NO_2_^−^ or NO_3_^−^.

In another study, a 3D nanoflower-like Cu_x_O/multilayer graphene composite was synthesized to measure NO_x_ gas molecules at room temperature [54]. The thermally expanded graphite (EG) was prepared by heating expandable graphite using intermittent microwave heating (IMH) method and activated with KOH to generate many moderate functional groups. The activated EG is labeled as aEG. The Cu_x_O/multilayer graphene composites (CuMGCs) were synthesized by vacuum assisted reflux method. Copper acetate and cetyltrimethylammonium bromide (CTAB) are incorporated into the layers of aEG by vacuum-assisted technique and, then, react with functional groups by exfoliation of aEG via reflux, which results in formation of 3D nanoflowers of Cu_x_O homogeneously grown on aEG.

**Figure 2:**
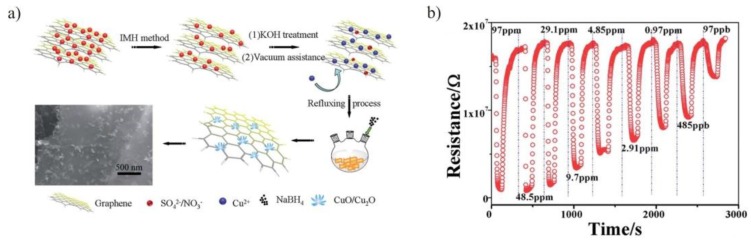
(**a**) Schematic illustration of the formation of Cu_2_O/MLG 3D nano-flowers; (**b**) Dynamic response-recovery curves of sensor device towards 97 ppm to 97 ppb NO_x_ at room temperature. {Reprinted with permission from [54], © 2014 Royal Society of Chemistry}.

The schematic illustration of the synthesis of CuMGCs is shown in Figure 2a. The sensitivity of CuMGCs sensor device was analyzed by exposing it to different concentrations of NO_x_ ranging from 97 ppm to 97 ppb at room temperature as shown in Figure 2b. It is evident from the curves that sensor response is highly dependent on gas concentration. The gas response and response time of sensor device towards 97 ppm were 95.1% and 10 s. At the lower concentration of 97 ppb (NO_x_), the sensor response was 27.1% with a response time of 59 s. The gas molecules extract electrons from CuMGCs when exposed to oxidizing gas NO_x_, which leads to a decrease in electron density and increases hole carriers on p-type semiconductor surface. This effect results in decreasing resistance of sensor material when NO_x_ gas molecules directly adsorb onto CuMGCs. The adsorption of NO_2_ on sensor material leads to NO_2_^−^ and adsorption of NO to NO^−^.

**Figure 3:**
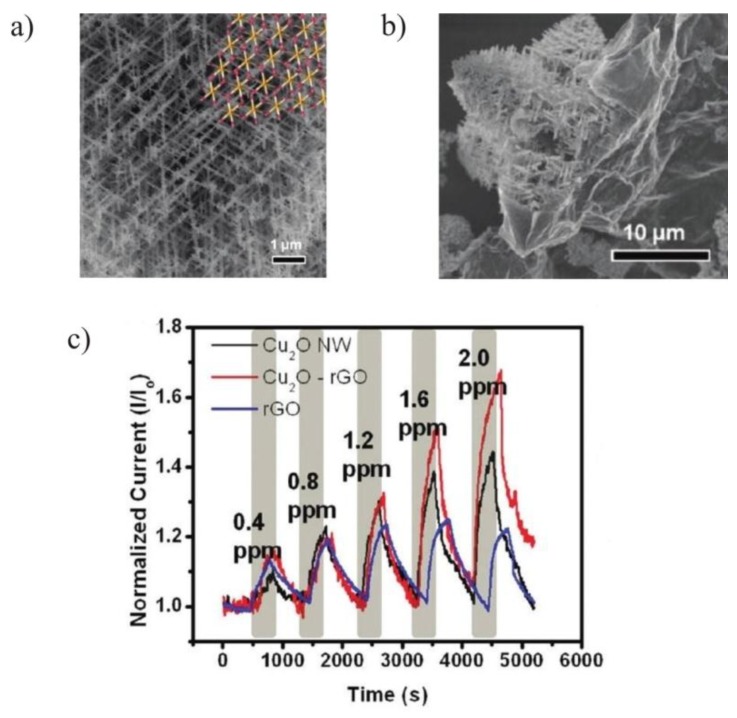
(**a**) FESEM image of Cu_2_O mesocrystal with octahedron morphologies comprised of branched nanowires; (**b**) FESEM image of Cu_2_O-rGO mesocrystal; (**c**) The response of rGO, Cu_2_O NW, and Cu_2_O-rGO sensor devices under increasing NO_2_ exposure. {Reprinted with permission from [55], © 2012 American Chemical Society}.

Cu_2_O NW-rGO composite mesocrystals were also synthesized by crystallizing copper (II) acetate in the presence of GO and o-anisidine under hydrothermal conditions [55]. These nanowire mesocrystals possess distinct octahedral morphologies and triangular external faces as evident from their field emission scanning electron microscopy image shown in Figure 3a. These octahedral mesocrystals are made of branched nanowires with diameters ranging from 80–110 nm. A small amount of GO (0.9 mg) is sufficient to initiate the formation of nanowire mesocrystals of Cu_2_O. GO interacts with Cu^2+^ ions during the *in situ* crystal growth and was subsequently reduced to rGO (under hydrothermal conditions) to form Cu_2_O mesocrystal interconnected via rGO network as shown in Figure 3b. Sensors devices based on rGO, Cu_2_O NW and Cu_2_O-rGO composite were fabricated. These sensors were exposed to different concentrations of NO_2_ ranging from 0.4–2 ppm as shown in Figure 3c. The sensing ability of Cu_2_O-rGO composite based sensor is greater in comparison to rGO and Cu_2_O NW based sensors. The calculated LOD of Cu_2_O-rGO based sensor is 64 ppb which is the best LOD in NO_2_ sensing at room temperature. The sensing mechanism for rGO-Cu_2_O composite can be explained as charge doping by adsorbing gas at sensor material which alters conductivity. The rGO prepared by chemical modification contains electron-withdrawing oxygen functionalities.

The rGO-Cu_2_O composite is a p-type semiconductor and releases its electrons to oxidizing gas NO_2_, which results in an increase in hole conductance. This composite is highly oriented and contains porous structure which gives a much larger surface accessibility in a 3D network. This effect results in increasing the sensor sensitivity towards adsorbed NO_2_ gas molecules.

Indium oxide (In_2_O_3_) was homogenously dispersed on rGO nanosheets by a hydrothermal method to synthesize a nanocomposite of In_2_O_3_-rGO [56]. The graphite powder was oxidized to produce GO and, then, hydrothermally treated with In(NO_3_)_3_ in the presence of hydrazine hydrate to obtain In_2_O_3_-rGO. The NO_2_ sensor was fabricated by coating a suspension of nanocomposite onto the IDEs. Different nanocomposite samples were prepared by varying rGO concentrations (0.72, 1.08, 2.16 and 4.32 wt%). The maximum sensor response of In_2_O_3_-rGO composite having 0.72 wt% of rGO was observed towards NO_2_ at room temperature. Co_3_O_4_ intercalated rGO based sensor showed a much higher response to NO_2_ at room temperature compared to rGO alone [57]. The increase in rGO concentration of composite leads to a decrease in sensor response due to a lower resistance of rGO in composite. Moreover, recovery becomes very difficult due to much strong adsorption of NO_2_ at defective sites of rGO. The enhanced sensor response can be explained by two possible mechanisms: increased surface area of rGO thick film by intercalation of Co_3_O_4_ and the Co^3+^-carbon coupling effect for rapid response. The in-between cobalt oxide (Co_3_O_4_) nanocrystals behave like nanopillars which leads to an extra macroporous structure between rGO layers. This results in better diffusion of gas molecules driven by capillary force which leads to enhanced response. The other possible mechanism is related to the coupling effect between Co and graphene, leading to enhanced oxygen reduction ability. The coupling between oxygen ions on graphene and Co makes Co-O a more ionic bond and results in Co^3+^ because extra interaction sites for NO_2_ molecules and electrons would be extracted indirectly from the p-type graphene through bridging oxygen. However, this composite based sensor showed a fast response and full recovery when exposed to methanol gas due to the interaction of gas molecules with sp^2^ carbon bonding sites. This interaction with gas molecules results in the release of electrons to graphene leading to a rise in resistance. The methanol gas molecules do not interact with oxygen containing defect sites because these species are negatively charged and reject electron donation from reducing species such as methanol.

Liu *et al*. prepared rGO-CNT-SnO_2_ hybrid from graphite powder, multiwalled carbon nanotubes (MWCNT) and tin chloride (SnCl_4_) by the hydrothermal method [58]. The aqueous dispersion of rGO-CNT-SnO_2_ hybrid was dip coated on ceramic substrate (already printed with gold electrodes) to fabricate NO_2_ sensor device. The incorporation of CNTs in rGO-SnO_2_ hybrid not only improve surface area of rGO by preventing its restacking but also improving electron transfer rate during gas sensing. The presence of SnO_2_ enhanced the recovery rate of hybrid sensor which was actually slow due to defects in the rGO structure. Thus, response and recovery time was greatly improved after incorporation of CNT and SnO_2_ in the rGO structure. The sensor based on rGO-CNT-SnO_2_ hybrid was used over a detection range of 1–100 ppm of NO_2_ gas at room temperature. The sensor exhibited a sensitivity of 2.53 at 5 ppm of NO_2_ gas with fast response/ recovery time of 8 s/77 s. The response and recovery time of rGO-CNT-SnO_2_ hybrid based sensor is better than previously proposed NO_2_ gas sensors [59,60,61,62,63]. Zhang *et al*. synthesized a nanocomposite based on SnO_2_-rGO by reacting aqueous dispersion of GO with Sn salt hydrothermally [60]. This nanocomposite was utilized to fabricate a sensor for detecting NO_2_ gas at low operating temperatures. The nanocomposite exhibited higher sensor response of 3.31 at 5 ppm of NO_2_ gas in comparison to response of 1.13 of rGO based sensor. A NO_2_ gas sensor has been developed on interdigitated Pt electrodes by synthesizing caesium doped graphene oxide (GO-Cs) [64]. The graphite was chemically oxidized to produce GO which was further doped with caesium by thermal solid-state reaction. The developed sensor based on GO-Cs exhibited higher sensor response to NO_2_ gas in comparison to pristine GO based sensors with the detection limit of approximately 90 ppb. Li *et al*. prepared three-dimensional (3D) SnO_2_/rGO composite by hydrothermal and lyophilization method to measure NO_2_ gas [65]. The 3D porous composites were synthesized by using different metal salts as precursors such as Sn^2+^ and Sn^4+^ and it was found that NO_2_ detection behavior of these composites was dependent on precursors. The Brunauer-Emmett-Teller method was used to analyze the surface area and porosity of 3D porous composite. It was found that surface area of porous composite prepared from Sn^4+^ precursor is large which is beneficial for gas sensing applications. The extremely large surface area of 3D porous composite made it possible to detect as low as 2 ppm of NO_2_ gas at low temperature.

A CO_2_-assisted thermal method was followed to synthesize iron oxide-graphene (Fe_2_O_3_-G) hybrid. This hybrid was further converted into paper-like vertically arranged Fe_2_O_3_-G nanosheets by a controlled magnetic field [66]. These paper-like materials were used to fabricate H_2_S gas sensor. Both vertically and horizontally aligned nanosheets were synthesized and used as sensing material for H_2_S, which showed that nanosheets with vertical arrangements are more beneficial than with horizontal arrangements in terms of sensitivity. In Fe_2_O_3_-G nanocomposite, the contents of both carbon and oxygen are decreased in comparison to that of GO. The decarboxylation reaction decreases carbon content, and dehydration is the cause for decreasing oxygen, which are the main reactions that occurred during formation of nanocomposite. The better sensitivity of vertically aligned Fe_2_O_3_/graphene is not only due to a large amount of uniformly distributed Fe_2_O_3_ on graphene sheets but also the special structure of vertically aligned nanosheets. This special structure provides a large contact area and less resistance which makes it very sensitive to H_2_S gas. In another study, a 3D array structure was synthesized from 1D SnO_2_ nanorods and 2D graphene sheets (SnO_2_-G 3D array structure) via a straightforward nanocrystal-seeds-directing hydrothermal method [67]. The growth of SnO_2_ nanorods on graphene sheets can be controlled by changing seeding concentration and temperature. Graphene sheets were synthesized by CVD method. The 3D array structures were synthesized in two steps: at first nanocrystal seeds were arranged hydrolytically on graphene sheets and then grown into a 3D structure hydrothermally. The gas sensing behavior of SnO_2_ is an adsorption-oxidation-desorption process which changes the electrical conductance of the sensing material. The electron depletion layer is generated on n-type SnO_2_ semiconductor due to the presence of surface oxygen species (O_2_^−^, O^−^, O^2−^) which increases surface potential barrier. These ions were consumed after exposure to reductive gas such as H_2_S which generates additional electrons resulting in increased material conductance. The sensing performances of SnO_2_-G 3D array structures are significantly better than SnO_2_ flowers due to the following reasons: (1) The uniform distribution and highly aligned nanorods of moderate diameter offer a large surface area which facilitate molecular adsorption, gas diffusion and mass transport; (2) The outstanding electrical conductivity and chemical sensitivity of graphene as a sensing platform lead to better gas sensing behavior. MalekAlaie *et al*. prepared molybdenum trioxide (MoO_3_) nanoparticle decorated GO for the detection of H_2_S [68]. At first, GO was synthesized from natural graphite by following modified Hummer's method and, then, it was reduced to rGO by exposure to hydrazine. A metal precursor ammonium molybdate tetrahydrate ((NH_4_)_6_Mo_7_O_24_) was used to decorate rGO by calcination at 550 °C in N_2_ environment to convert Mo to MoO_3_. MoO_3_-rGO composite was spin coated between Pt electrodes on alumina substrate to fabricate a sensor device. Sensor response was investigated by adding different wt% of metal precursor in different concentrations of graphene oxide suspensions. The composite of 3 wt% of MoO_3_ and 5 mg/mL of GO suspension exhibits higher sensitivity at different concentrations (50–500 ppm) of H_2_S at 160 °C. The operating temperature of MoO_3_-rGO is lower in comparison to pristine rGO.

## 5. Conclusions

A comparison of graphene based gas sensors is described in Table 1. According to these data, the NO_2_ gas sensor based on epitaxial graphene exhibited the lowest LOD of 0.6 ppt (detection range of 0.2 ppb–1 ppm) in comparison to all other graphene based sensors fabricated for NO_2_ gas detection (LOD in ppb range). The sensitivity of the sensor device towards NO_2_ down to the sub-ppt level is due to the activation of high binding energy adsorption centers by an annealing process. This annealing process removed contaminants on the sensor surface which remained on the chip after fabrication. The introduction of functional oxygen groups to graphene by ozone treatment increases the sensor response to 17%. The fabrication of highly oriented rGO microbelts for ammonia gas showed the highest detection range of 10–1000 ppm at operating temperatures of 40–100 °C. Ethanol based CVD grown graphene nanomesh (G_ethanol_) expressed the lowest LOD of 160 ppb which is attributed to highly disordered sp^3^ hybridized carbon atoms, a higher number of edge defects, and functionalized oxygen groups. The reduction of graphene oxide by hydrogen plasma exhibited a detection range of 0–1500 ppm with a sensor response of 71% to CO_2_ gas, which is due to the presence of more sp^2^ crystalline interaction sites.

A comparison of graphene polymer hybrid based gas sensors has been presented in Table 2. A gas sensor made of G-PEDOT:PSS composite film exhibited the highest detection range (5–1000 ppm) to NH_3_ gas. However, rGO-PPy is capable of detecting the lowest concentration of NH_3_ gas (1 ppb) with a response/recovery time of 1.4 s/76 s. The higher sensing performance of rGO device was due to the intrinsic properties of graphene sheets as well as adsorbed PPy molecules. The aniline reduced GO showed higher sensor response of 37% towards NH_3_ gas but its response time is very high (18 min). PPy-rGO nanofibers exhibited a detection range of 1–50 ppm to NO_2_. The rGO based NO_2_ gas sensor which was fabricated on electrospun Nylon-6 nanofiber scaffold is able to detect the lowest concentration down to 0.25 ppm in comparison to all other NO_2_ gas sensors based on graphene polymer hybrids.

**Table 1:** Gas sensors based on graphene. G = Graphene; FET = field effect transistor; rGO = reduced graphene oxide; RT = room temperature; ppm = parts per million; ppt = parts per trillion.

Sr. No.	Graphene Hybrid	Type of Sensor	Target Gas	Temperature (°C)	Detection Range (ppm)	LOD	Response (Sensitivity)	Response Time	Recovery Time	Reference
1	Epitaxial-G	Chemiresistor	NO_2_	RT	0.2 ppb–1 ppm	0.6 ppt	−	−	−	[13]
2	G-ozone treated	Chemiresistor	NO_2_	RT	0.2–200	1.3 ppb	17% (200 ppm)	−	−	[14]
3	G-exfoliated	Chemiresistor	NO_2_	RT	100 and 500	−	9% (100 ppm)	−	−	[15]
4	G-nanomesh	Chemiresistor	NO_2_/NH_3_	RT	1–10/5–100	15/160 ppb	4.32%/0.71%	5–7 min/−	−	[16]
5	rGO	FET	NH_3_	40–100	10–1000	−	1%–38%	∼9–12 min	−	[17]
6	G-microfiber	Optical	NH_3_	−	0–360	0.3 ppm	−	∼0.4 s	−	[18]
7	rGO	Chemiresistor	CO_2_	RT	0–1500	−	71% (1500 ppm)	∼4 min	∼4 min	[19]
8	Graphene sheets	Conductivity sensor	CO_2_	22–60	10–100	−	9%–26%	8 s	10 s	[20]
9	rGO	Chemiresistor	H_2_	30–300	200	−	6%–17% (200 ppm)	∼11 s	∼36 s	[21]

**Table 2:** Gas sensors based on graphene polymer hybrids. G = Graphene; rGO = reduced grapheme; poly(3,4-ethylenedioxythiophene): poly(styrenesulfonate)(PEDOT:PSS); polypyrrole (PPy); poly(methyl methacrylate) (PMMA); RT = room temperature; PANI = polyaniline; PPD = p-phenylenediamine; DMMP = dimethyl methyl phosphonate.

Sr. No.	Graphene Hybrid	Type of Sensor	Target Gas	Temperature	Detection Range (ppm)	LOD	Response (Sensitivity)	Response Time	Recovery Time	Reference
1	G-PEDOT-PSS	Chemiresistor	NH_3_	RT	5–1000	<10 ppm	1.2%–18.9%	∼3 min	5 min	[22]
2	PMMA-rGO	SPR	NH_3_	RT	10–100	−	−	<1 min	−	[23]
3	PPy-rGO	Chemiresistor	NH_3_	RT	5 ppb–100 ppm	−	22% (100 ppm)	−	134–310 s	[24]
4	PPy-rGO	Chemiresistor	NH_3_	RT	1 ppb–50 ppm	−	2.4% (1 ppb)	1.4 s	76 s	[25]
5	rGO-PANI	Chemiresistor	NH_3_	RT	20–50	−	37.1% (50 ppm)	18 min	∼2 min	[26]
6	rGO	Chemiresistor	NH_3_	RT	5–50	−	10.7%–47.6%	18 min	−	[27]
7	rGO-PPy	Chemiresistor	NH_3_	RT	3–500		1.105%–34.73%	400–147 s	−	[28]
8	PEDOT-rGO	Chemiresistor	NO_2_	RT	500 ppb–20 ppm	−	−	−	−	[29]
9	rGO nanofiber	Chemiresistor	NO_2_	RT	0.25–4.5	−	7% (0.25 ppm)	−	−	[30]
10	rGO-PPy	Chemiresistor	NO_2_	RT	1–50	−	−	−	−	[31]
11	G-Nylon 6	Chemiresistor	N(CH_3_)_3_	RT	23–230 mg/L	0.39 mg/L	7.38% (45 mg/L)	100 s	−	[32]
12	rGO-PPD	Chemiresistor	DMMP	RT	5–80	−	14.5% (80 ppm)	18 min	6 min	[33]

**Table 3:** Gas sensors based on graphene metal/metal oxide hybrids. G = Graphene; rGO = reduced grapheme; eG = epitaxial grapheme; SLG = single-layer grapheme; GNR = graphene nanoribbons, RT = room temperature, Pt = Platinum, Pd = Palladium, Au = Gold, NPs = nanoparticles, NG = nitrogen doped graphene, PPy = polypyrrole, P3HT = poly(3-hexylthiophene), NW = nanowires, Cs = caesium, QDs = quantum dots.

Sr. No.	Graphene hybrid	Type of Sensor	Target Gas	Temperature (°C)	Detection Range (ppm)	LOD	Response (Sensitivity)	Response Time	Recovery Time	Reference
1	PMMA-Pd NP-SLG	Chemiresistor	H_2_	RT	0.025%–2%	−	66.37% (2% H_2_)	1.81 min	5.52 min	[34]
2	Pd-GNR	Chemiresistor	H_2_	RT	30–1000	−	4.5% (1000 ppm)	60 s	90 s	[35]
3	Pd-WO_3_-rGO	Chemiresistor	H_2_	RT to 250	20–10,000	−	−	<1 min	<1 min	[36]
4	G-Pt/Pd	Chemiresistor	H_2_	RT	6–40,000	1 ppm	36% (10,000 ppm (1%))	3 min	1.2 min	[37]
5	Pt-NG/Pt_3_Fe-NG	Chemiresistor	H_2_	RT	1%–4%	−	47%/35% (4%)	320 s/300 s	−	[38]
6	G-ZnO	Chemiresistor	H_2_	150	10–500	10 ppm	3.5 (200 ppm)	22 s	90 s	[39]
7	rGO-SnO_2_ QDs	Chemiresistor	H_2_/LPG	200/250	100–500	−	89.3%/92.4% (500 ppm)	∼50–160 s/90–135 s	∼100–160 s/90–160 s	[40]
8	rGO-Pt	Chemiresistor	H_2_/NH_3_/NO	RT	200–1000	−	14%/8%/10% (1000 ppm)	2–6 min	2–6 min	[41]
9	GO-Au NPs	Optical	H_2_/CO/NO_2_	150	100 & 10,000/10,000/1	−	0.0007–0.004/–/0.0004	1–2/–/12 s	2-4/–/15 s	[42]
10	Ag-ZnO-rGO	Chemiresistor	C_2_H_2_	150	1–1000	1 ppm	21.2 (100 ppm)	25 s	80 s	[43]
11	SnO_2_-rGO	Chemiresistor	C_2_H_2_	RT	10–2000	−	2.19%–9.72%	107–146 s	95–141 s	[44]
12	ZnO-G	Chemiresistor	CH_2_O	RT	180 ppb–9 ppm	180 ppb	52% (9 ppm)	36 s	−	[45]
13	G-ZnFe_2_O_4_	Chemiresistor	(CH_3_)_2_CO	275	1–1000	−	1.5–9.1	0.73–10.59 s	14.75–26.16 s	[46]
14	G-ZnO	Chemiresistor	C_2_H_5_OH	300	10–50	−	9–90	−	−	[47]
15	G-mica	FET	NH_3_	RT and 100	20–100	−	−	−	−	[49]
16	SnO_2_-G	Chemiresistor	NH_3_	RT	10–50	−	15.9% (50 ppm)	<1 min	<1 min	[50]
17	TiO_2_-PPy-G	Chemiresistor	NH_3_	RT	1–200	−	102.2% (50 ppm)	36 s	16 s	[51]
18	ZnO-GO	Conductivity sensor	CO/NH_3_/NO	RT	−	−	24.3/24/3.5% (22/1/5 ppm)	5/6/25 min	2–5/2–3 min	[52]
19	Co(OH)_2_-rGO	Chemiresistor	NO_x_	RT	970 ppb–97 ppm	0.97 ppm	70% (100 ppm)	−	−	[53]
20	CuO-G	Chemiresistor	NO_x_	RT	97 ppb–97 ppm	97 ppb	95.1% (97 ppm)	9.6 s	−	[54]
21	rGO-Cu_2_O	Chemiresistor	NO_2_	RT	0.4–2	64 ppb	67.8% (2 ppm)	−	−	[55]
22	In_2_O_3_-G	Chemiresistor	NO_2_	RT	5–100	−	8.25 (30 ppm)	4 min	24 min	[56]
23	Co_3_O_4_-rGO	Chemiresistor	NO_2_	RT	60	−	−	−	−	[57]
			CH_3_OH	RT	300–1000	−	−	∼1–2 min	∼1–2 min	
24	rGO-CNT-SnO_2_	Chemiresistor	NO_2_	RT	1–100	−	2.53 (5 ppm)	8 s	77 s	[58]
25	SnO_2_-rGO	Chemiresistor	NO_2_	50	0.5–500	−	3.31 (5 ppm)	135 s	200 s	[60]
26	GO-Cs	Conductometric	NO_2_	RT	0.18–12.2	90 ppb	0.7%–39.6% (0.18–12.2 ppm)	4 min	−	[64]
27	SnO_2_-rGO	Chemiresistor	NO_2_	22–70	14–110	2 ppm	1.079 (100 ppm) at 55 °C	−	373 s	[65]
28	Fe_2_O_3_-G	Optical (CL)	H_2_S	190	15	<10 ppm	450 au (15 ppm)	500 µs	30 s	[66]
29	G-SnO_2_	Chemiresistor	H_2_S	260	1–50	−	2.1 (1 ppm)	5 s	10 s	[67]
30	MoO_3_-rGO	Chemiresistor	H_2_S	160	50–500	−	4120 (50 ppm)	60 s	120 s	[68]

Table 3 highlights the sensing performance of graphene metal/metal oxide based gas sensors. The hydrogen gas sensor fabricated by incorporating Pd-WO_3_ nanostructures in GO and partially reduced graphene oxide (PRGO) expressed a detection range of 20–10,000 ppm with a response/recovery time of <1 min. Whereas, a higher sensor response of 89.3% towards hydrogen gas was observed by a sensor device based on rGO-SnO_2_ QDs at an operating temperature of 200 °C. An optical type H_2_ sensor exhibited excellent response/recovery time of 1–2 s/2–4 s at an operating temperature of 150 °C. TiO_2_-PPy-G based NH_3_ gas sensor showed a higher sensor response of 102.2% with an excellent response/recovery time of 36 s/16 s at room temperature in comparison to all other NH_3_ gas sensors. A chemiresistor type acetylene gas sensor based on Ag-ZnO-rGO exhibited higher sensor response (21.2%) and excellent response/recovery time (25 s/80 s) but it requires an operating temperature of 150 °C. 3D nanoflower of CuMGC exhibited excellent sensor response (95%) but its response time (9.6 s) is a little bit slower than the rGO-CNT-SnO_2_ based sensor having a response time of 8 s. whereas, Cu_2_O-rGO nanowires mesocrystals expressed the lowest LOD of 64 ppb to NO_2_. A chemiresistor based on MoO_3_-rGO composite exhibited a higher sensor response of 4120 to 50 ppm of H_2_S with a response/recovery time of 60 s/120 s at a low operating temperature of 160 °C. A 3D array structure (1D SnO_2_ and 2D graphene sheets) based H_2_S sensor exhibited better response/recovery time but the operating temperature of this sensor device was 260 °C.

Graphene materials and their composites offer an attractive way to fabricate sensor devices for different gases and vapors. These graphene based sensors exhibited excellent performance compared to all other sensors in terms of sensitivity, limit of detection and reversibility. These sensors have the advantage over other sensors due to their low energy consumption and ability to operate at room temperature. The unique properties of graphene such as high surface area, mechanical strength and better electrical/temperature tolerance make this material a promising candidate for developing gas sensors which can operate in extreme conditions.

Apart from a large number of advantages, there are still some issues which need to be addressed for fabricating graphene sensors on a commercial scale. Highly sophisticated techniques are required for the fabrication of ultrathin graphene layers. The simple and widely used fabricating techniques such as spin coating, drop casting or inkjet printing cannot be applied for synthesizing ultrathin graphene layers. Moreover, the number of graphene sheets is also very difficult to control.
